# Disentangling brain vasculature in neurogenesis and neurodegeneration using single-cell transcriptomics

**DOI:** 10.1016/j.tins.2023.04.007

**Published:** 2023-05-18

**Authors:** Elizabeth E. Crouch, Tara Joseph, Elise Marsan, Eric J. Huang

**Affiliations:** 1 Department of Pediatrics, University of California San Francisco, San Francisco, CA 94143, USA; 2 Eli and Edythe Broad Center of Regeneration Medicine and Stem Cell Research, University of California San Francisco, San Francisco, CA 94143, USA; 3 Department of Pathology, University of California San Francisco, San Francisco, CA 94143, USA; 4 Pathology Service (113B), San Francisco Veterans Administration Health Care System, San Francisco, CA 94121, USA

## Abstract

The vasculature is increasingly recognized to impact brain function in health and disease across the life span. During embryonic brain development, angiogenesis and neurogenesis are tightly coupled, coordinating the proliferation, differentiation, and migration of neural and glial progenitors. In the adult brain, neurovascular interactions continue to play essential roles in maintaining brain function and homeostasis. This review focuses on recent advances that leverage single-cell transcriptomics of vascular cells to uncover their subtypes, their organization and zonation in the embryonic and adult brain, and how dysfunction in neurovascular and gliovascular interactions contributes to the pathogenesis of neurodegenerative diseases. Finally, we highlight key challenges for future research in neurovascular biology.

## Vasculature and brain health

The discovery of the circulation by William Harvey (1578–1657) is one of the greatest milestones in modern medicine [1]. Harvey’s discoveries were later amplified by the 17th century Dutch anatomist Frederik Ruysch (1638–1731), whose work, sometimes described as ‘Rembrandts of anatomical preparation’, recognizes the foundational importance of blood vessels [2]. Nearly four centuries after Harvey’s and Ruysch’s discoveries, the vasculature is increasingly recognized to impact brain function in health and disease across the life spectrum [3–6]. Both genetic and sporadic disorders of the vasculature during development can severely influence long-term neurological function. Genetic mutations in subunits of type IV collagen, the main component of vascular basement membranes, cause porencephaly in neonates and cerebrovascular disease in adults [7–9]. Separately, about 20% of preterm babies born before 30 gestational weeks (GWs) experience brain hemorrhage that can cause permanent neurological dysfunction and even death [10–12]. This condition underscores the urgent need to investigate mechanisms that govern angiogenesis in the prenatal human brain [13,14]. Later in life, brain arteriovenous malformations (AVMs) are a leading cause of stroke in young people [15,16], and based on studies in both humans and mouse models, dysfunctions in brain vascular cells are now recognized to contribute to neurodegeneration and brain aging [17–22]. Finally, neural stem cell-derived endothelial cells are integral to the growth of glioblastoma, an aggressive and lethal form of brain cancer [23,24]. In sum, neurovascular interactions are not only critical for normal development, but also catalyze brain pathologies.

This review focuses on recent discoveries on the molecular and cellular mechanisms utilized by the vasculature to regulate brain development and degeneration. We focus mainly on publications that leverage single-cell transcriptomics, including single-cell RNA-seq (scRNA-seq) and single-nucleus RNA-seq (snRNA-seq), to uncover the detailed molecular underpinnings of brain vascular components. We also discuss how this information advances current understanding of the intersection of the brain vasculature with neurogenesis during brain development and in neurodegenerative diseases.

## Cellular composition of the brain vasculature

Compared with endothelial cells in the peripheral organs, brain endothelial cells exhibit distinct features, including reduced transcytosis, absence of fenestrations, presence of tight junctions, and extremely low permeability, which contribute to the blood–brain barrier (BBB) [25–29]. While these features insulate the brain from potentially toxic substances from the systemic circulation, they present a significant challenge to drug delivery. By leveraging single-cell transcriptomic and epigenomic technologies, several recent studies have provided critical insights into the cellular composition and identity of the brain vasculature.

### Endothelial and mural cell subtypes in mouse brain

The availability of vascular cell subtype-specific reporter mice offers a distinct advantage in the isolation and characterization of these cells using single-cell transcriptomics. To this end, a recent study used *Pdgfrb*-GFP;*Cspg4*-DsRed to isolate mural cells [30] and another employed *Tagln*-Cre;*R26*-stop-tdTomato for smooth muscle cells and *Pdgfra*-H2BGFP for perivascular cells from embryonic mouse brain [31]. A separate approach used the *Tie2-GFP* reporter to isolate and characterize the transcriptomes and epigenomes of endothelial cells from brain, liver, lung, and kidney of postnatal day 7 (P7) mice [32]. Although *Tie2-GFP* is not expressed in all endothelial cells, this study identifies arterial endothelial cells that express *Bmx*, *Efnb2*, *Vegfc*, and *Sema3g*, capillary endothelial cells that express *Mfsd2a* and *Tfrc*, and venous endothelial cells that express *Nr2f2* (Table 1) [32]. Two additional endothelial cell subtypes, mitotic endothelial cells and tip cells, can be detected in P7 mouse brain, suggesting that these dynamic endothelial cells are primarily present during early postnatal brain development [32]. Finally, a third approach utilized cell type-specific markers and fluorescence-activated cell sorting (FACS) to isolate endothelial cells, mural cells, neural cells, and microglia from wild-type mouse brain at embryonic day 14.5 (E14.5) [33] or endothelial cells from various organs in 8-week-old mice [34]. Although fewer endothelial cells are present in the embryonic mouse brain, heterogeneity of endothelial cells exists as early as E14.5 [33].

**Figure 1.**
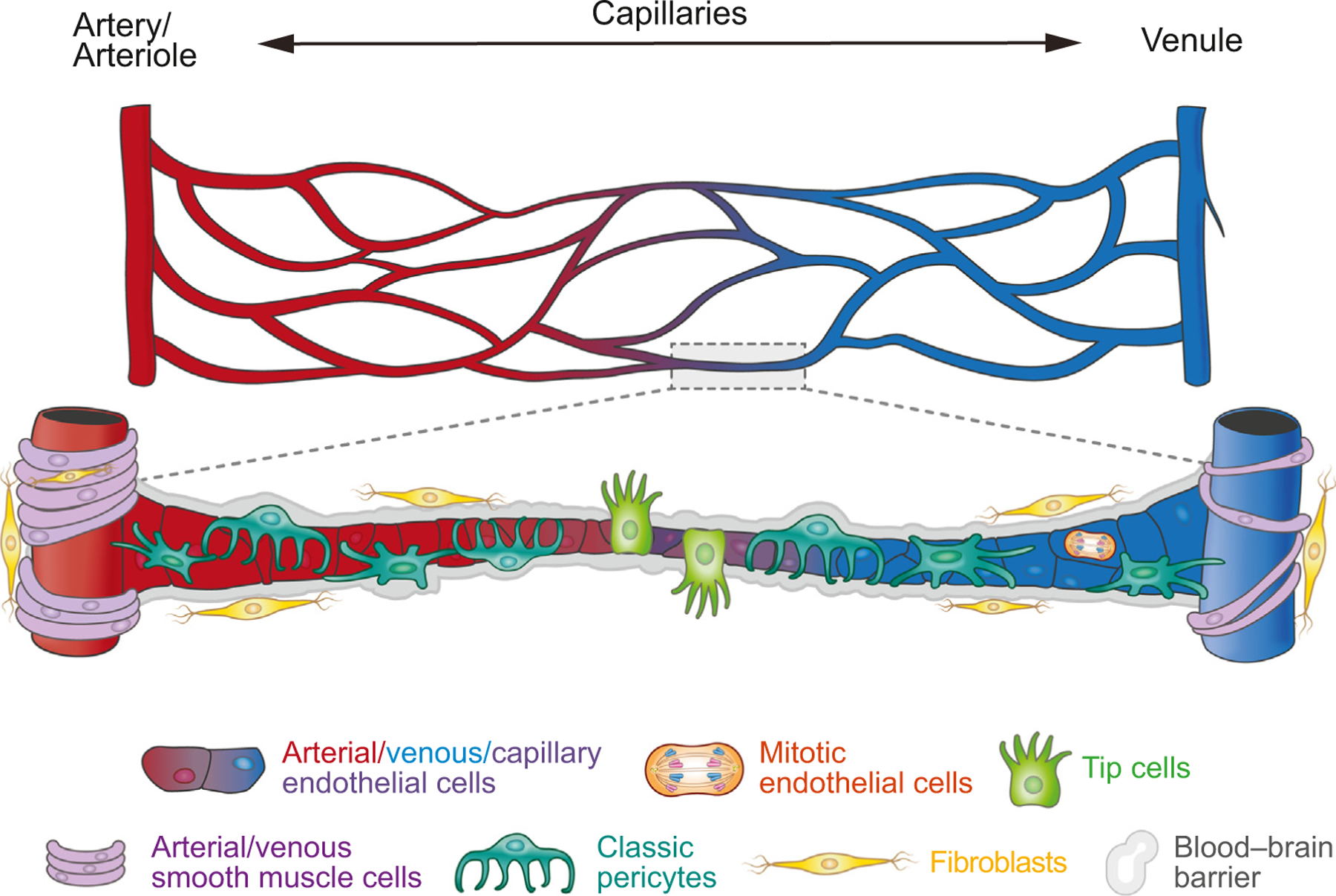
Zonation and cellular composition of the arteriovenous axis in the prenatal human brain vasculature. A simplified, schematic illustration of the arteriovenous axis of the vasculature in the developing brain and some of the primary associated cell types. Single-cell transcriptomics of brain endothelial cells and mural cells from the second trimester shows cell type-specific gene expression patterns that support the presence of zonation in the brain vasculature at the early stage of prenatal human brain development [13]. These datasets also identify distinct subtypes of endothelial cells, including arterial endothelial cells, venous endothelial cells, and capillary endothelial cells, and two specialized endothelial cells, tip cells and mitotic endothelial cells, which are more common in the developing blood vessels in the ventricular and subventricular zones in the embryonic brain. Similarly, single-cell transcriptomics reveals subtypes of mural cells, including arterial and venous smooth muscle cells, pericytes, and fibroblasts. The interactions between endothelial cells and mural cells regulate the formation of blood–brain barriers.

Given the critical function of the brain vasculature in providing nutrients and removing waste, it has been postulated that endothelial cells may transition gradually along the arteriovenous axis [5]. In support of this idea, initial endothelial cell-specific transcriptomic analyses revealed a gradual transition of gene expression profiles from arterial to capillary to venous endothelial cells indicative of cellular zonation (Figure 1). Further refinement showed that capillary endothelial cells could be divided between cells simultaneously expressing some arterial and some venous genes [31,32]. Consistent with morphological observations, mitotic cells also expressed capillary/venous and venous genes, suggesting that proliferation in endothelial cells largely arises towards the venous end of the endothelial spectrum [35–37]. In the adult mouse brain, tip and mitotic cells could no longer be identified [31]. Interestingly, arterial endothelial cells show preferential expression of transcription factors whereas transmembrane transporters are more enriched in capillary and venous endothelial cells. These cell type-specific gene expression findings have been corroborated by other studies and may contribute to BBB transport in capillary and venous locations [29,38,39].

Similar to endothelial cells, scRNA-seq analyses of mural cells also demonstrated zonation along the arteriovenous axis. Evidence supporting this idea includes the expression of *Cnn1*, which encodes the actin-binding protein calponin 1 (CNN1), in arterial smooth muscle cells (aSMCs) and the expression of *Acta2* and *Tagln*, which encode α-smooth muscle actin (ACTA2) and smooth muscle protein 22-α (TAGLN), respectively, in arteries and arterioles (Table 2). The expression of these genes in smooth muscle cells is conserved in both mice and zebrafish and is weak in venous smooth muscle cells (vSMCs). Interestingly, expression of these genes has not been detected in pericytes, indicating an abrupt transition from arteriolar smooth muscle cells (aaSMCs) to pericytes along the arteriole–capillary boundary. Like endothelial cells, the zonal distribution of mural cells is underscored by the gene expression and physiological differences. Pericytes in adult mouse brain show abundant expression of SLC, ABC, and ATP transporters [31], which differs from lung pericytes, suggesting that brain pericytes may exhibit organotypic specialization that contributes to the formation and function of the BBB. Preliminary results suggest that the ATP transporter *ATP13A5* is abundantly expressed in pericytes, similar to *KCNJ8* and *VTN* (Table 2) [40]. In addition, several studies have shown that pericytes of different morphologies, such as ensheathing, mesh, and thin strand, can be identified along the vascular tree contributing to BBB formation [41,42], although the molecular signatures of these pericyte subtypes remain unclear. Finally, scRNA-seq analyses also identified a unique cluster of fibroblasts, which are thought to be distinct from mural cells [43,44], that exhibit enriched expression of genes encoding fibrillary and non-fibrillary collagens, collagen-modifying enzymes, and proteins involved in collagen fibril spacing, including proteoglycan lumican (*Lum*) and decorin (*Dcn*) (Table 2). Gene ontology annotations of these fibroblast-associated genes suggest that these cells could be involved in the formation of extracellular matrix (ECM) as well as cell adhesion and migration.

### Endothelial and mural cell subtypes in human brain

Compared with other CNS cell types such as neurons, astrocytes, and oligodendroglia, vascular cells constitute a minority. With the advent of sophisticated tissue dissociation reagents, cell type-specific markers, and FACS-based protocols, there has been tremendous progress in isolating and enriching vascular cells from prenatal and adult human brain tissues [13,15,17,18]. For instance, the methodology of vessel isolation and nucleus extraction for sequencing (VINE-seq) combines a sucrose gradient and FACS to remove myelin debris and enrich for vascular cells, respectively, to isolate vascular cells. A recent study conducted VINE-seq on hippocampal and frontal cortex tissue from individuals with normal cognition or with Alzheimer’s disease (AD) and subjected the isolated vascular cells to snRNA-seq [18]. Another approach, called blood vessel enrichment (BVE), utilizes dextran-based density ultracentrifugation to enrich vascular cells from fresh and frozen human brain tissues for single-cell transcriptomic analyses [17]. A third approach uses microdissection of brain vasculature followed by enzymatic digestion of neurosurgical specimens from patients undergoing lobectomy for epilepsy [15]. Finally, to study angiogenesis in the prenatal human brain, our group has modified a previously published FACS-based scheme [45,46] with CD31 and ANPEP antibodies to label endothelial and mural cells, respectively, in prenatal human brain from the second trimester [13].

Collectively, these approaches show that the majority of endothelial cells are capillary, with subsets of arterial and venous endothelial cells. Interestingly, tip cells – a specialized subtype of endothelial cells that guide the leading edge of angiogenesis – represent about 5–15% of endothelial cells in the prenatal human brain during the second trimester (Figure 1). In contrast, in the adult brain, only a very small cluster (~0.1%) of tip cells are detected in one study [18], and no tip cells are detected in two other studies [15,17]. Another subtype, called mitotic endothelial cells, is detected only in the brain vasculature in prenatal human brain and not in adult brain. Besides these differences, there are notable similarities in the human brain vascular cell repertoire over time. With respect to mural cells, the three aforementioned studies identify two subtypes of smooth muscle cells, aSMCs and vSMCs [15,17,18]. In terms of the developmental trajectory of pericytes, classic pericytes are present in the developing brain vasculature as early as the second trimester [13]. In adult brain vasculature, two clusters of pericytes are detected: transport pericytes (T-pericytes), which are enriched with small-molecule transmembrane transporters such as the GABA transporter *SLC6A1* and the glutamate transporter *SLC1A3*; and matrix pericytes (M-pericytes), which are enriched with ECM organization [17,18]. Finally, these studies also identified distinct clusters of fibroblasts, including perivascular fibroblasts, which show enriched expression of ECM proteins, and meningeal fibroblasts, which show enriched expression of solute transporters. These fibroblasts are present as early as the second trimester [13], suggesting their engagement in angiogenesis in the prenatal human brain (Figure 1).

**Figure 2.**
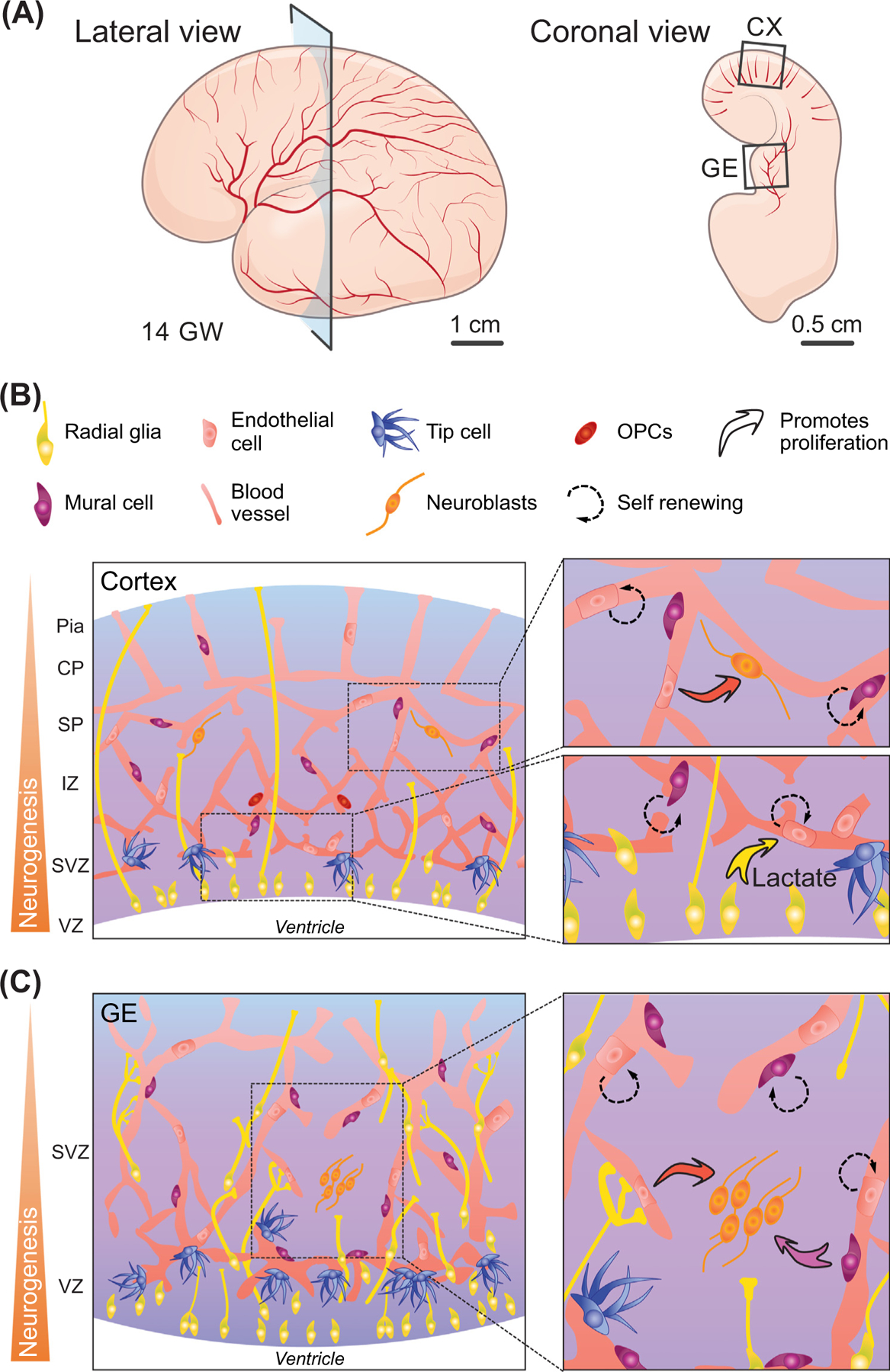
Neurovascular interactions in neurogenic niches during embryonic brain development. (A) Schematic diagrams showing a lateral view and a coronal hemisection of a prenatal human brain at 14 gestational weeks (GWs), highlighting the developing cortex (CX) and ganglionic eminence (GE). (B) In the developing cortex (pallium), neural progenitors closely interact with the vasculature in the ventricular and subventricular zones (VZ/SVZ) in the developing cortex. In relatively avascular regions, neural progenitors generate lactate via anaerobic metabolism to promote vascular growth. (C) In the GE (subpallium), the vasculature is much more dense in the VZ/SVZ and the vascular cells promote neurogenesis. Key cell types in the VZ/SVZ in the developing cortex and GE are highlighted in (B). Abbreviation: OPC, oligodendroglial precursor cell.

The rich single-cell transcriptomic datasets of the human brain vasculature offer ample opportunities to define the transcriptional landscape of human brain vascular cells and for comparisons with similar datasets in the mouse brain. In a study on E14.5 mouse brains, vascular cells showed heterogeneity but no clear subtypes [33]. This, however, may be due to the relatively limited number of cells analyzed in the study. In the prenatal human brain, by contrast, the vasculature was found to contain all of the major subtypes of endothelial and mural cells in the second trimester, in both the developing cerebral cortex and the germinal matrix [13]. Interestingly, the blood vessels outside the neurogenic niches in the prenatal human brain show endothelial zonation along the arteriovenous axis, whereas blood vessels in neurogenic niches such as the ventricular zone (VZ) and subventricular zone (SVZ) contain all of the vascular cell subtypes arranged in a mosaic pattern (Figure 2). Blood vessels in the VZ and SVZ contain more mitotic endothelial cells and tip cells and are intertwined with neural progenitors [13]. Vascular cells in the adult human brain exhibit well-established arterial-capillary-venous zonation and the genes involved in vascular zonation differ significantly from those in adult mouse brain (Tables 1 and 2). In addition, vascular cells of the human brain may have further specializations based on metabolic characteristics [17,18].

For instance, a subtype of arterial endothelial cells in adult human brain expresses *TXNIP*, which encodes thioredoxin-interacting protein implicated in glucose metabolism and oxidative stress, suggesting that these endothelial cells may have a different metabolic state [15]. Additional evidence supporting the unique metabolism of human vascular cells is the expression of *HIGD1B*, which encodes hypoxia inducible domain family member 1B, in more than 90% of pericytes in the adult human brain, again pointing to the unique capabilities of human brain vascular cells in oxygen sensing [15]. The differences between mouse and human endothelial cells suggest that similar investigations into the molecular signatures of vascular cells in other species, such as zebrafish [47], might provide additional insights regarding species-specific transcriptomic profiles.

Finally, the second-trimester vascular cell scRNA-seq dataset also permits the first query into the ontogeny of vascular cells in the human brain. RNA velocity analysis projects that the endothelial cell trajectory begins with proliferative and venous cells, progresses through a capillary intermediate, and ends with tip and arterial cells separately [13]. This prediction is consistent with previous morphological and transcriptomic studies in the embryonic and early postnatal mouse brain [32,35–37,48]. The ontogeny of mural cells is less defined. RNA velocity analysis of second-trimester human brain mural cells suggests that smooth muscle cells are progenitors that may give rise to pericytes and parenchymal fibroblasts [13]. The precise developmental staging of embryonic mouse brain endothelial and mural cells has not been determined, but postnatal analysis of KCNJ8^+^ mural cells shows this population as progenitors for smooth muscle cells [49]. In agreement with this lineage relationship, cardiac coronary artery smooth muscle cells are derived from Notch3^+^ pericytes in the embryonic mouse [50]. Further delineation of cell fate and lineage in vascular cells is likely to provide insights into the pathogenesis of congenital vascular malformations and have implications in regeneration and therapeutics.

### Crosstalk between endothelial and mural cells and the formation of the BBB

The intricate interactions between endothelial cells and pericytes are critical to the development of the BBB [38,39,51]. Signaling pathways that regulate these interactions include ephrins, Notch, angiopoietins, and transforming growth factor beta (TGFβ) [39]. For cellular recruitment, endothelial cells secrete platelet-derived growth factor subunit B (PDGFB) to promote the recruitment and maintenance of pericytes and vascular integrity [52–55]. In the developing mouse brain, endothelial cells from the perineural vascular plexus migrate into the neural parenchyma at E9.5 and are closely followed by PDGFRβ^+^ pericytes [30,56]. Mice with homozygous deletion in *Pdgfrb* (*Pdgfrb*^−/−^) or carrying the PDGFB retention motif knockout that disrupts PDGFB binding to heparan sulphate proteoglycans (*Pdgfb*^*ret*/*ret*^) reveal the critical role of pericytes in regulating BBB-specific gene expression in endothelial cells and in the induction of polarization of astrocyte end feet around blood vessels. Even *Pdgfrb*^+/−^ mice show reductions in brain microcirculation, including diminished brain capillary perfusion and cerebral blood flow (CBF), and pronounced BBB breakdown during brain aging [51]. To enable BBB formation, mural cells in embryonic mouse brain express vitronectin, which interacts with endothelial integrin alpha5 [33,57]. In the human brain, both CD31^+^ and PDGFRβ^+^ vascular cells are present throughout the brain regions by the start of the second trimester [13]. Based on CellChat, a cellular interaction bioinformatics tool, ECM proteins such as collagen, laminin, and fibronectin are among the most dominant signaling pathways that regulate crosstalk between CD31^+^ endothelial cells and PDGFRβ^+^ mural cells in the second trimester. Traditional angiogenic signals like PDGFB, vascular endothelial growth factor (VEGF), ANGPT, insulin-like growth factor (IGF), and epidermal growth factor (EGF) are also predicted to regulate these interactions. The most abundant growth factor-related pathway is midkine (MDK), which promotes angiogenesis and is structurally related to pleiotrophin [58,59]. In support of this idea, MDK promotes vascular tube formation in 3D Matrigel assays using primary endothelial cells and mural cells from the second-trimester human brain [13].

### The intersection of angiogenesis and neurogenesis in prenatal brain development

Classic quail-chick transplantation chimeras show that blood vessels grafted with neural tissue form structural, functional, and histochemical features of the BBB [60]. These results usher many intriguing studies aimed at uncovering the mechanisms regulating the mutual interactions between angiogenesis and neurogenesis in embryonic and adult brain [61]. In the developing mouse cortex, the pattern of nascent vasculature appears to correlate with the initiation of neurogenesis [62]. This vascular niche continues to interact with neural stem cells in the SVZ in adult mouse brain [63–65]. Given the critical role of the vasculature in providing oxygen, it is interesting to note that angiogenesis establishes distinct hypoxic and perivascular niches, where the dividing apical progenitors in the VZ are preferentially localized in the avascular regions whereas the basal progenitors and oligodendroglial precursor cells (OPCs) preferentially contact the vasculature [66]. Interestingly, the mutual interactions between angiogenesis and neurogenesis are further supported by single-cell transcriptomic data showing that radial glia produce a high level of lactate via anaerobic metabolism to promote vascular growth [67]. By inference, intermediate progenitor cells, OPCs, and basal progenitors would be most likely to possess different metabolic profiles given their preferential association with perivascular regions and branch points [66,68].

In subpallial regions, physical contact between the vasculature and neural progenitors also regulates key aspects of differentiation. Vascular filopodia in the ganglionic eminences contact radial glia in the human and mouse prenatal brain, which elongates their cell cycle and favors neuronal differentiation [69]. By contrast, radial glia extend fibers that are anchored to local blood vessels, and disruption of this interaction decreases the number of interneurons produced by these cells [70]. In the hindbrain, deletion of NRP1 in endothelial cells showed a premature decline in both NPC activity and hindbrain growth downstream of precocious cell cycle exit, premature neuronal differentiation, and abnormal mitosis patterns [71]. In the embryonic spinal cord, Sema3C in motor neurons interacts with PlexinD1 in endothelial cells to repel vascular entry to the dorsal column; disruption of this interaction disrupts motor neuron exit from the spinal cord [72]. Collectively, these findings highlight a combination of physical, metabolic, and molecular signals that orchestrate a complex series of coordinated stages of angiogenesis and neurogenesis.

### Vascularizing brain organoids

Cortical brain organoids offer a promising model system to interrogate the interactions between human vascular and neural cells. Since vascular and neural cells are derived from different germ layers, several strategies have been developed to implant primary brain organoids into the mouse brain [73], to add endothelial or pericytes derived from human pluripotent stem cells (hPSCs) [74–76], or to fuse separate hPSC-derived vascular and neural organoids [77,78]. These studies support the overarching principle that neurovascular coupling is critical to promote neurogenesis, angiogenesis, astrogliogenesis, and the formation of the BBB. In addition, neurovascular assembloids offer a feasible model to investigate the mechanism of viral tropism – for instance, of severe acute respiratory syndrome coronavirus 2 (SARS-CoV-2) – via neurovascular borders. However, one major limitation of these organoid models is the lack of information regarding the developmental trajectories of diverse vascular cells in the neurogenic niche and how these vascular cells promote neurogenesis. To address these challenges, in a study from our group we transplanted FACS-purified human brain endothelial and mural cells onto cerebral organoids and allowed the organoids to develop further for 2 weeks more [13]. This approach shows that the majority of the transplanted endothelial cells acquire tip cell markers, whereas the majority of the mural cells display smooth muscle cell markers. These results suggest that the neurogenic environment in the cerebral organoids is likely to influence cell-fate specification in these vascular cells. Interestingly, the presence of vascular cells significantly increases the number of RBFOX3^+^ (NeuN^+^) and BCL11B^+^ (CTIP2^+^ layer 5–6 excitatory neurons) in the cerebral organoids and reduces cellular stress [13]. Collectively, these results support the critical role of neurovascular interactions in angiogenesis and neurogenesis.

## Neurovascular interactions in neurodegenerative diseases

### Imaging-based studies of vascular dysfunction in AD

In addition to its role in neurogenesis, several lines of evidence indicate that dysfunction in the brain vasculature makes critical contributions to age-related neurodegenerative diseases including AD [19,79], although the exact mechanisms remain unclear. For instance, studies using spin-labeled magnetic resonance imaging (MRI) suggest that individuals with mild cognitive impairment (MCI) or early AD develop reduced CBF in brain regions such as the posterior cingulate gyrus and the precuneus [80]. However, another study questioned the utility of regional CBF as a sole indicator, and argued that the combination of an entorhinal cortical atrophy score and CBF is a better predictor of progression from MCI to AD [81]. Other parameters that show brain vascular dysfunction in AD patients include impaired cerebrovascular reactivity in response to a CO_2_ inhalation challenge and impaired neurovascular coupling in response to neuronal stimulation [82]. Another potential contributing factor is the presence of cerebral amyloid angiopathy (CAA), which is characterized by the deposition of Aβ in small- to-medium-sized blood vessels [83]. However, the formal cause–effect relationship connecting CAA to vascular dysfunction in AD remains unclear (see Outstanding questions).

AD patients with CAA have a higher risk of developing microbleeds, which are caused by loss of BBB integrity and blood leakage into brain parenchyma. Pathologically, microbleeds are characterized by the presence of hemosiderin-laden macrophages in the perivascular spaces in the white matter. The exact causes of these abnormal vascular manifestations remain unclear. However, *APOE4* carriers without dementia appear to have more impairments in these parameters than non-*APOE4* carriers [84], suggesting that APOE4 may disrupt glia–vasculature homeostasis to facilitate the vascular pathology in the white matter [85,86]. In addition, hypertension positively correlates with microbleed prevalence [87]. Much of the vascular pathology in AD patients can be recapitulated in animal models where transgenic expression of the human mutant *APP* gene leads to perivascular accumulation of Aβ [88]. In addition, several blood-derived proteins, including fibrinogen, IgG, and albumin, are identified in the blood vessels of AD mouse models, leading to vascular leakage and damage to endothelial cells and pericytes [89].

### Single-cell transcriptomics of vascular cells in AD

**Figure 3.**
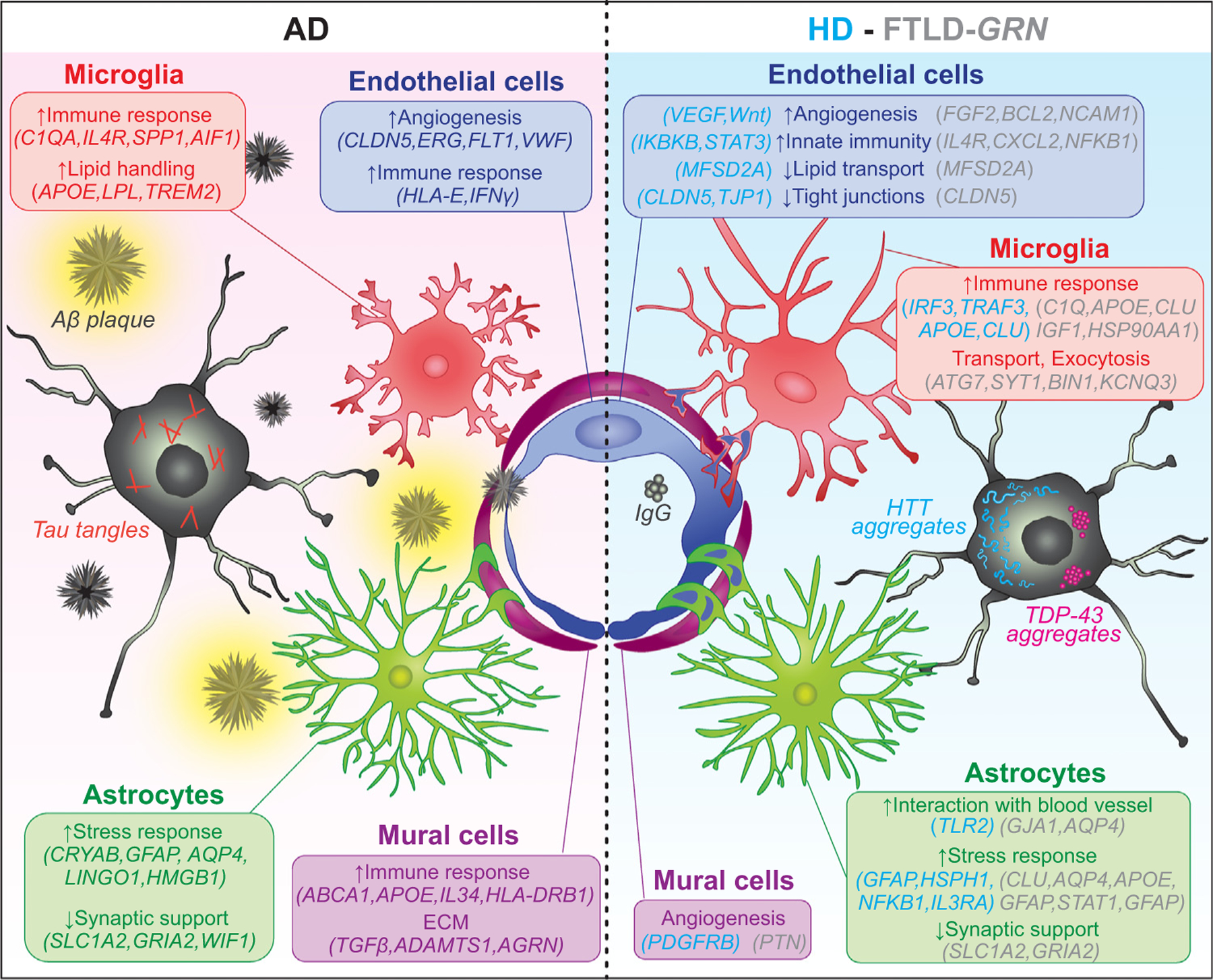
Perturbations of glial–vascular interactions in neurodegenerative diseases. Single-cell transcriptomics studies using postmortem brain tissues from patients with Alzheimer’s disease (AD) [18], Huntington’s disease (HD) [17], or frontotemporal lobar degeneration caused by dominant mutations in the *GRN* gene (FTLD-*GRN*) [103,104] reveal significant alterations in the transcriptomes of endothelial cells and mural cells (including pericytes). The transcriptomic changes in the vascular cells in these diseases implicate increased angiogenesis, activation of an immune response, and perturbations of tight junctions, lipid transport, and the extracellular matrix (ECM). In addition, these datasets reveal robust transcriptomic changes in microglia and astrocytes that interact with the vascular cells. Microglia and astrocytes in these neurodegenerative diseases show transcriptomic changes implicating activation of an immune response, increased lipid transport and exocytosis, increased stress response, increased interaction with blood vessels, and reduced synaptic support.

What are the molecular mechanisms that drive the vascular abnormalities in AD? Analyses of vascular cells from AD brain tissue, compared with those from age-matched controls, show proportional reductions in endothelial cells, pericytes, smooth muscle cells, and perivascular fibroblasts, suggesting a widespread loss of vascular cells [90]. Relatedly, vascular cells in AD brains do not exhibit region-specific differences in relative abundance and transcriptomic profiles in the hippocampus and frontal cortex [18]. Furthermore, the reduced endothelial cell density leads to morphological changes in the capillaries that resemble ‘string vessels’ previously described in AD, ischemia, and irradiation [91]. Analyses of the differentially expressed genes (DEGs) in brain vascular cells from AD patients reveal that the most robust changes in DEGs are detected in the mural cells, although the exact impact of these transcriptomic changes at the morphological and functional levels remains unclear. Interestingly, snRNA-seq studies in AD brains show increased expression of angiogenic growth factors and their receptors in the endothelial cells (Figure 3) [92–95]. Furthermore, endothelial cells in *APOE4* carriers exhibit transcriptomic changes suggesting prominent interferon-mediated inflammation. Somewhat surprisingly, direct comparisons reveal that the transcriptomic changes in brain endothelial cells in human AD brain show minimal overlap with those in a transgenic AD mouse model that overexpress APP with the London (V717l) and Swedish (K670M/N671L) mutations (*Thy1-hAPP*^*Lon;Swe*^ mice) [18]. In sum, studies on human AD brains support the notion that vascular cells in AD exhibit a wide range of abnormalities, including defects in zonation and regional specification, that could contribute to the functional perturbations observed in imaging studies.

Several genome-wide association studies (GWASs) have identified genes that increase the risk of AD [96–99]. Cell type-specific transcriptomic profiling and epigenetic mapping in the promoter–enhancer regions show that many of these AD GWAS genes have critical functions in microglia, the brain’s innate immune cells [100]. Interestingly, based on the vascular cell transcriptomic dataset [18], several AD GWAS genes, including many that have been implicated in immune functions in mice, are also expressed in human vascular cells. For instance, *APOE*, which has been linked to microglia and astrocytes, also exhibits robust expression in smooth muscle cells (Figure 3). Other GWAS genes, including *ABCA1*, *FHL2*, *HESX1*, and *IL34*, are enriched in fibroblasts. These results underscore the species-specific differences in the expression of AD GWAS genes in different brain vascular cell types. They also raise the intriguing hypotheses that vascular cells in the human brain could be targeted by immune cells and that aberrant activation of immune-related pathways may facilitate brain aging and increase the risk of AD.

### Single-cell transcriptomics of vascular cells in Huntington’s disease

Previous studies have implicated several brain vascular phenotypes in Huntington’s disease (HD), including increased BBB permeability, increased small vessel density, altered vascular morphology and cerebral blood volume, and activation of pericytes [101]. These changes can be detected before the neurodegenerative phenotypes in both mouse HD models and presymptomatic HD patients [102]. However, the exact mechanisms for these vascular phenotypes remain unclear. By comparing snRNA-seq datasets of vascular cells between control and HD patients, a recent study showed that endothelial cells in HD display significant downregulation of *MFSD2A* [17], a lipid transporter that restricts caveola-mediated transcytosis (Figure 3) [29]. Furthermore, endothelial cells in HD show upregulation of genes that are implicated in angiogenesis, endothelial cell migration, the activation of VEGF and Wnt signaling, and downregulation of the tight junction proteins CLDN5 and TJP1 (ZO-1). Interestingly, the transcriptomes of endothelial cells in HD also reveal the enrichment of many innate immune activation genes, including *IKBKB*, *IRF2*/*3*, and *STAT3*. Similar increases in these innate immune genes can be detected in microglia and astrocytes in HD, suggesting that there may be coordinated transcriptomic and functional perturbations to glia–vasculature homeostasis. Histopathological validations show that many reactive astrocytes and microglia appear to have engulfed vascular cells in HD [17]. Interestingly, similar observations are reported in recent snRNA-seq analyses of the thalamocortical circuit in frontotemporal lobar degeneration caused by dominant mutations in the *GRN* gene (Figure 3) [103,104]. Collectively, these results suggest that disruption of glia–vasculature interactions could be a shared feature in neurodegenerative diseases.

## Concluding remarks and future perspectives

The advent of single-cell transcriptomics has uncovered the molecular underpinnings of vascular cell subtypes and how they are organized during prenatal brain development and in the adult brain. By directly comparing the transcriptomes of these vascular cells and their neighboring cells, including glia and neurons, progress has been made towards a better understanding of neurovascular interactions in health and diseases. These advances mark the beginning of new chapters in research to tackle many challenges in neurovascular biology, including in addressing the mechanisms regulating the formation and functions of neurovascular units [105], in engineering *in vitro* models to recapitulate the mutual interactions between the tumor microenvironment and the vasculature [106–108], and in reprogramming vascular cells to promote tissue regeneration [109]. Furthermore, understanding of immune–vascular interactions could have major impacts on the delivery of small molecule- and cell-based therapies to combat neurodevelopmental and neurodegenerative diseases.

## Figures and Tables

**Table 1.** Transcriptomic profiles of endothelial cell subtypes

Species	Arterial endothelial cells^a^	Capillary endothelial cells^a^	Venous endothelial cells^a^	Mitotic endothelial cells^a^	Tip cells^a^	Refs
Mouse	*BMX*, *CDKN1C*, *COL18A1*, *CSCL12*, *DKK2*, *EFNB2*, *EGFL8*, *ELN*, *FBLN2*, *FBLN5*, *GJA4*, *GKN3*, *HSPA1A*, *HTRA1*, *LGFBP3*, *LTBP4*, *MGP*, *MSX1*, *PTPRR*, *S100A10*, *SEMA3G*, *SERPINF1*, *SMAD6*, *SYT15*, *TM4SF1*, *VEGFC*	Capillary-A: *ABHD17C*, *BCR*, *CYP2D22*, *CDKN2B*, *CXCR4*, *DDC*, *FGFBP1*, *GPR85*, *HBA-A1*, *HBA-A2*, *HBB-BS*, *HBB-BT*, *KCNJ2*, *LYPD1*, *PCX*, *PRR18*, *SCGB3A1*, *SEMA3C*, *SLC1A1*, *SLC7A3*, *SLCO1A4*, *SPOCK2*, *STRA6*, *TMC7*, *VSTM2B* Capillary-V: *AMT*, *ATIC*, *ATP1B1*, *BEST1*, *CBLN2*, *CHN2*, *DBH*, *E130012A1Rik*, *FAM69B*, *FTH1*, *GPATCH4*, *GM6792*, *GM11496*, *KLK8*, *LMNTD1*, *LRRC55*, *METTL1*, *MOB3B*, *MYC*, *NDNF*, *NKD1*, *PRDX3*, *RBP1*, *SRM*, *UNG*	*ADH1*, *ADGRG6*, *ARRDC4*, *BMP2*, *CAR14*, *CCL19*, *CLEC14A*, *COL15A1*, *CRISPLD1*, *CUTAL*, *EFCC1*, *FMO1*, *GPIHBP1*, *GPR182*, *LBP*, *MAFB*, *MMRN1*, *MYOF*, *NBL1*, *PLAGL1*, *NRP2*, *PAQR5*, *PCP4L1*, *TXNIP*	*ANLN*, *AURKB*, *BIRC5*, *BUB1*, *BUB1B*, *CCNB1*, *CDCA5*, *CENPE*, *CEP55*, *DEPDC1A*, *DIAPH3*, *DLGAP5*, *FAM64A*, *FAM83D*, *HMMR*, *KIF20A*, *KIF20B*, *KIF23*, *KIF4*, *KIFC1*, *MXD3*, *NUF2*, *PLK1*, *SHCBP1*, *SKA1*, *UBE2C*	*ADM*, *APOD*, *ANGPT2*, *CHST1*, *CLEC1A*, *CMKLR1*, *GM8817*, *HECW2*, *KCNA5*, *KCNE3*, *MADCAM1*, *MCAM*, *NOXO1*, *OAF*, *PCDH17*, *PDE4B*, *PIEZO2*, *PLAUR*, *PPM1J*, *PRRG3*, *SCN1B*, *SERPINE1*, *SIRPA*, *SMOC2*, *TRP53i11*	[32]
	*BMX*, *EFNB2*, *GKN3*, *SEMA3G*, *VCAM1*, *VEGFC*	*MFSD2A*, *SLC16A1*, *TFRC*	*NR2F2*, *SLC16A1*, *SLC38A5*, *TFRC*, *VCAM1*	N/A^b^	N/A	[31]
Human	Arteriole: *ARL15*, *CLDN5*, *DKK2*, *FLT1*, *VEGFC*	*ABCB1*, *ATP10A*, *CMTM8*, *MFSD2A*, *NPIPB5*, *SLC7A5*, *SYNE1*	Venule: *ADGRG6*, *AFF3*, *SLC2A1*, *TSHZ2*	N/A	N/A	[17]
	Type 1: *HSPA1A*, *INTS6*, *JUNB* Type 2: *ARL15*, *MECOM*, *VEGFC* Type 3: *MGP*, *PECAM1*, *TXNIP*	*MFSD2A*, *RGCC*, *SLC3A2*, *SRARP*	Venous: *ACKR1*, *IL1R1*, *TSHZ2* Venule: *ATP10A*, *MFSD2A*, *TMEM132C*	N/A	N/A	[15]
	*ALPL*, *PLCG2*, *VEGFC*	*ABCG2*, *MFSD2A*, *SLC39A10*, *SLC7A5*	*IL1R1*, *NR2F2*	N/A	*HSPH1*, *LAMB1*, *PLAUR*	[18]
	*C10orf10*, *C12orf75*, *CXCL12*, *FBLN5*, *GJA4*, *HEY1*, *ID1*, *KCTD12*, *UNC5B*	*CD27*, *CSRP2*, *HES1*, *MFSD2A*, *PON2*, *SLC39A10*, *SLCO1A2*	*IFI27*, *IL1R1*, *LY6E*, *PMAIP1*, *PRCP*, *RAMP3*, *RPS2*, *RPS23*, *S100A10*, *TSHZ2*	*BIRC5*, *CENPF*, *HMGB2*, *HMGN2*, *NUSAP1*, *TOP2A*, *TUBA1B*, *UBE2C*	*ADM*, *ANGPT2*, *APLN*, *COL9A3*, *CTGF*, *CXCR4*, *LXN*, *LY6H*, *TIMP1*	[13]

a Arterial endothelial cells carry oxygenated blood away from the heart with pulsatile flow [110]. Capillary endothelial cells are thought to be the primary site of the BBB and oxygen exchange [29]. Venous endothelial cells carry deoxygenated blood at lower pressure towards the heart and may interact with peripheral immune cells in the brain like other organs [110,111]. Tip cells are an endothelial subtype unique to the angiogenic vasculature that sit at the end of a vascular tube and extend filopodia to determine vascular growth [112]. Mitotic endothelial cells are dividing cells.

b N/A, not available.

**Table 2.** Transcriptomic profiles of mural cell subtypes, fibroblasts, and fibromyocytes

Species	Smooth muscle cells^a^	Classic pericytes^a^	Mitotic mural cells^a^	Fibroblasts^a^	Fibromyocytes^a^	Refs
Mouse	General: *PDLIM3* aSMCs: *ACTA2*, *CNN1*, *EGR1*, *FOS*, *FOSB*, *JUN*, *JUNB*, *MYL9*, *MYH11*, *TAGLN* aaSMCs: *ACTA2*, *MYL9*, *MYH11*, *TAGLN* vSMCs: *ABCC9*	*ABCC9*, *ANPEP*, *ATP13A5*, *BAIAP3*, *CD248*, *CSPG4*, *DES*, *HIGD1B*, *IFITM1*, *KCNJ8*, *MCAM*, *PDGFRB*, *RGS5*, *S1PR3*, *VTN*	N/A^b^	*COL1A1*, *COL12A1*, *COL3A1*, *COL5A1*, *COL8A2*, *DCN*, *LUM*, *MMP2*, *PDGFRA*	N/A	[31,40]
Human	aSMCs: *ACTA2*, *KCNAB1*, *MYH11*, *NTN4* vSMCs: *CD74*, *HBB*, *HLA-DRA*, *MCAM*, *MYOCD*	Type 1: *CA4*, *ITM2A*, *SLC38A5*, *SPOCK2* Type 2: *CERS6*, *DAB1*, *GRM8*, *SHISA6*	N/A	Type 1: *ABCA10*, *ANXA2*, *CEMIP*, *COL12A1*, *FBLN1*, *GFPT2*, *RNF220*, *SVIL* Type 2: *MYRIP*, *SLC13A3*, *TP63*, *TRPM3* Type 3: *EYS*, *KCNMA1*, *SLC4A4*, *SLIT2*	N/A	[17]
	General: *CNN1*, *MYH11*, *TAGLN* Type 1: *DES*, *MYL9* Type 2: *GLA*, *GRID2*, *RERGL* Type 3: *HSPA1A*, *HSPA1B*, *HSPA6* Type 4: *APOE*, *NDUFA44L2*, *SLC38A11* Type 5: *C11orf96*, *CARMN*, *RN7SK* Type 6: *MT1M*, *MT1X*, *MT2A* Type 7: *CCL2*, *IL6*, *RGS16*	*ABCC9*, *ATP1A2*, *HIGD1B*, *KCNJ8*	N/A	*ALDH1A1*, *ALDH1A2*, *APOD*, *IGF2*, *DCN*, *MGP*, *PTGDS*	Type 1: *IGFBP5*, *KCT2*, *RARA* Type 2: *ALDH1A1*, *CCL19*	[15]
	aSMCs: *ACTA2*, *TAGLN* aaSMCs: *CTNNA3*, *SLIT3* vSMCs: *MCAM*	General: *ABCC9*, *PTN* Transport pericytes: *CTDSPL*, *PTPRK*, *SLC1A3*, *SLC12A7*, *SLC20A2*, *SLC6A1*, *SLC6A12*, SLC6A13 Matrix pericytes: *ADAMTS1*, *COL4A1*, *COL4A2*, *COL4A3*, *COL4A4*, *CRISPLD2*, *LAMA4*	N/A	Meningeal: *SLC13A3*, *SLC22A23*, *SLC24A3*, *SLC26A2*, *SLC26A7*, *SLC35G1*, *SLC38A2*, *SLC39A11*, *SLC4A4*, *SLC41A2*, *SLC47A1*, *SLC7A2*, *SLC9B2* Perivascular: *ABCA10*, *ABCA11*, *ABCA6*, *ABCA8*, *ABCA9*, *SLC7A11*	N/A	[18]
	*ACTA2*, *CTGF*, *HSPA2*, *IGFBP2*, *MYL9*, *SDC2*, *TAGLN*, *TPM1*, *TPM2*, *VIM*	*ABCC9*, *HSPA1A*, *HSPA1B*, *IFITM1*, *JUNB*, *KCNE4*, *KCNJ8*, *PRKCDBP*, *SLC6A12*, *SPARCL1*, *SPON2*	*CCNB1*, *CDK1*, *CENPF*, *HIST1H4C*, *HMGB2*, *KPNA2*, *NUSAP1*, *PTTG1*, *TOP2A*, *UBE2C*	*CLU*, *COL1A1*, *COL3A1*, *CTHRC1*, *CXCL12*, *CYP1B1*, *NDUFA4*, *OGN*, *PTGDS*, *VCAN*	N/A	[13]

a Smooth muscle cells are contractile and present primarily in the larger brain blood vessels. Pericytes are mural cells of blood microvessels, which are likely to play a critical role in BBB formation and may also play a role in vascular tone [38,39,113–116]. Mitotic mural cells are dividing cells. Parenchymal fibroblasts are present in the perivascular space and may contribute to scar formation, but their functions during homeostasis are not fully clear [117].

b N/A, not available.

## Highlights

Single-cell transcriptomics of brain vascular cells reveals distinct subtypes of endothelial and mural cells, as well as transcriptomic profiles defining their organization and zonation in the embryonic and adult brain.

Mutual interactions between angiogenesis and neurogenesis promote the formation of the blood–brain barrier and coordinate the proliferation, differentiation, and migration of neural progenitors.

A full repertoire of endothelial and mural cell subtypes can be identified in the ventricular and subventricular zones of the cerebral cortex and germinal matrix during embryonic development, as early as the second trimester in humans.

Vascularized brain organoids recapitulate many key features of neurovascular coupling during angiogenesis and neurogenesis, including the cell-fate specification of vascular cells, cortical neuron differentiation, and the reduction of cellular stress.

Single-cell transcriptomics reveals profound dysfunction in neurovascular and gliovascular coupling as key features in several neurodegenerative diseases.

## Outstanding questions

What are the vascular stem cells for endothelial and mural cells? What are the mechanisms that control their transitions into different subtypes? Can these vascular stem cells be leveraged and harnessed for regenerative medicine?

Do neural stem cells and vascular stem cells in neurogenic niches such as the VZ and SVZ share the same identity? If so, are there subsets of these stem cells that are shared? How do these shared characteristics vary according to developmental time or location?

What are the molecular mechanisms that control the ontogeny and specification of endothelial and mural cell subtypes? How do endothelial and mural cells establish the zonation within the brain vasculature? Does perturbation of vascular cell subtypes contribute to vascular malformation?

What are the cellular and molecular mechanisms that regulate the development, establishment, and functions of neurovascular units?

How do perivascular macrophages and other immune cells interact with and regulate the homeostasis of vascular cells? Why do microglia, astrocytes, and vascular cells exhibit coordinated activation of immune-related genes during neurodegeneration and brain aging? Is dysfunction in these cells an active driver of neurodegeneration?

How can *in vitro* models be engineered to fully recapitulate neurovascular interactions during brain development and degeneration?
